# “Treat Me Like a Person”: Unveiling Healthcare Narratives of Muslim Women who Wear Islamic Head Coverings Through a Poststructural Narrative Study

**DOI:** 10.1177/08445621241258871

**Published:** 2024-06-03

**Authors:** Rezwana Rahman, Jennifer Lapum, Nadia Prendergast

**Affiliations:** 1 Daphne Cockwell School of Nursing, 7984 Toronto Metropolitan University, Toronto, ON, Canada

**Keywords:** Canadian health systems, Canadian health care, cultural issues, nursepatient relationships, women's health, transcultural nursing

## Abstract

**Background:**

In Canada, the healthcare experiences and needs of Muslim women who wear Islamic head coverings are conflated with the larger Muslim community who do not wear Islamic head coverings. Understanding their specific and unique preferences and challenges is essential for tailoring care and improving healthcare encounters.

**Purpose:**

The study purpose is to explore the healthcare encounters of Muslim women wearing Islamic head coverings in Canada, focusing on how discourse influences their narratives.

**Methods:**

A postructuralist narrative methodology was used to understand how power, knowledge, language, and discourse impacted their experiences. Semi-structured interviews were conducted with eight Muslim women. Narrative analysis was used to dissect stories and the way these stories were told.

**Results:**

Five themes were identified, including:

1. The Fingerprint: Highlights the importance of recognizing individual identities to provide personalized care.
2. The Membrane: Examines how societal biases and assumptions permeate healthcare professionals and impacts care.
3. The Heartbeat: Reveals the immediate emotional and physical responses that reflect systemic challenges within healthcare encounters.
4. Unseen: Emphasizes the lack of acknowledgement experienced by Muslim women related to their healthcare preferences and/or needs.
5. Heard: Encompasses instances where Muslim women feel recognized by their healthcare provider; contrasts Theme #4.

**Conclusion:**

This research emphasizes the diverse experiences of Muslim woman who wear an Islamic head covering and the need for healthcare professionals to move away from a one-size-fits-all approach and instead, provide care that respects the unique preferences amongst this diverse group.

## Background and purpose

There are many assumptions and stereotypes about Muslim women particularly those who wear Islamic head coverings such as the hijab. Embedded in Islamophobia and the hypervisibility of wearing an Islamic head covering, they are misperceived as both oppressed and lacking agency (Saleh et al., 2023). Drawing upon the work of Said (1978), these assumptions and stereotypes work to create a sense of othering that Muslim women are different from non-Muslim people. This othering discourse further acts to reinforce bias, discrimination and racism against Muslim women. In addition to being viewed as the “strange other”, they are often categorized as one homogenous group (Razack, 2008). These dominant discourses shape knowledge and power; additionally, these social discourses also infiltrate healthcare.

Research on Muslim women's experiences in healthcare remains limited in Canada and other Western countries. In current healthcare research, existing literature often addresses the broader experiences of Muslim women, regardless of whether they wear Islamic head coverings or not (Alzghoul et al., 2021; Nur, 2014). This generalization eclipses the specific challenges faced by Muslim women who's Islamic head coverings subject them to discrimination and misunderstandings within the healthcare system because of their visibility.

Current literature also tends to focus on specific geographical spheres or experiential categories, such as women who grew up in Eastern parts of the world (Simpson & Carter, 2008) or women specifically in receipt of perinatal care (Alzghoul et al., 2021). Alzghoul and colleagues found that Muslim women prefer female healthcare providers and that most responded well to their accommodation needs for modesty. However, requests for female providers sometimes resulted in delayed care (Vu et al., 2016). The narrow geographical and specialty focus of existing literature misses the broader range of healthcare encounters Muslim women encounter daily. Others found that Muslim women who wear a hijab reported negative healthcare interactions in which they were discriminated against (Nur, 2014). Simpson and Carter's research highlighted how Muslim women felt like a stranger in the healthcare system and how their encounters were negatively influenced by power relations. Their work highlights the gap in the understanding how the visibility of Muslim women impact their healthcare encounters.

Existing literature, although unintentional, may reinforce stereotypes that exists among Muslim women who wear an Islamic head covering. Specifically, Tackett et al. (2018) highlighted the importance of using interpreters in healthcare encounters when caring for Muslim women, which pushes forward a simplistic and unnuanced view of their healthcare preferences. The simplicity of this idea reinforces existing assumptions that Muslim women have low literacy rates (Rahman, 2023).

In the sphere of healthcare, the pursuit of inclusivity and cultural safety remain an ongoing necessity. Yet, experiences of Muslim women who wear Islamic head coverings are often concealed within this landscape. What is problematic is that existing literature often groups Muslim women as a whole and does not focus specifically on those who wear an Islamic head covering. This is a significant gap considering that Muslim women who wear an Islamic head covering cannot go unnoticed in their healthcare encounters and are a particularly stigmatized population in Canada. Additionally, a focus on practical approaches to healthcare negates a contextual understanding of Muslim women's experiences and how power and discourse shape their healthcare encounters. Subsequently, this fails to address the critical aspects of cultural safety and inclusivity within the healthcare system.

This research aims to fill the significant gaps listed above by focusing exclusively on Muslim women across Canada who wear Islamic head coverings. It intends to reveal the intricate encounters, societal nuances, and individual stories that shape the healthcare experiences of Muslim women who wear Islamic head coverings. Using these stories, this article strives to recalibrate the healthcare landscape, dismantling stereotypes and biases about this group of women so that care is empathetic, inclusive, and tailored to the diverse perspectives of Muslim women who wear Islamic head coverings. The purpose of this research was to delve into the encounters of Muslim women who wear Islamic head coverings within the Canadian healthcare system as patients. The study's questions were: How do Muslim women who wear Islamic head coverings narrate their experiences of healthcare encounters as a patient in Canada? How does discourse shape their narratives?

## Methods and procedures

### Theoretical framework

In this study, poststructuralism was employed to explore the intersections between language, discourse, knowledge and power (Mohammed et al., 2015), and how these shape identities and perceptions (Heslop, 1997). Grounded primarily in the perspectives of Foucault, this study aligns with three poststructuralist principles, as outlined by Holmes and Gagnon (2018). This framework challenges the notion of absolute truths, placing emphasis on how certain perspectives influence the comprehension of various phenomena. For instance, the word “hijab” held a very positive meaning for the first author of this article (who is a Muslim woman who wears an Islamic head covering) but may be perceived to be oppressive by others. Therefore, this method is most appropriate for this study as it specifically illuminates the diverse experiences and interpretations of Muslim women who wear an Islamic head covering, which is needed when understanding the multiplicity of their realities and experiences.

This framework also advocates for the interrogation of established knowledge, seeking not to destruct but to deconstruct existing structures. Poststructuralism does not seek to obliterate personal realities, but rather to unravel the intricate threads of how these truths have evolved and been shaped over time. This is exceptionally important to consider as religious identities intersect with cultural and social domains of life. Through poststructuralism, the researcher is allowed to delve into how the intersectionality of identities influences healthcare encounters of Muslim women who wear an Islamic head covering.

Poststructuralism also underscores the significance of informed critique, resisting dominant discourses through a nuanced understanding of the intricate relationship between knowledge and power (Holmes & Gagnon, 2018). A pertinent example of this was an article written by a Canadian doctor characterizing the hijab as a symbol of oppression (Emil, 2021); in response, there were various critiques which prompted its retraction. By examining the participants’ stories through poststructuralism, the study delves into how women navigate and resist dominant narratives and discourses that marginalize or oppress them.

In the realm of poststructuralism, language shapes subjective realities under the influence of prevailing power dynamics. Notably, the media frequently portrays Muslim women as marginalized or as “others” thus deeply embedding certain perceptions of Muslim women within societal consciousness (Hoodfar, 2003). Within the intricate interplay of knowledge and power, certain forms of knowledge often take precedence (i.e., images propagated by the media), establishing dominant discourses while subduing others. This phenomenon is evident in the discourse surrounding Islamic head coverings, encapsulating a diverse array of perspectives that significantly mold societal perceptions. This research, framed within the poststructuralist paradigm and employing narrative inquiry, provides a platform for Muslim women who wear Islamic head coverings to articulate their experiences. By challenging established ideologies, this study aims to dismantle entrenched power dynamics and reshape societal discourses concerning Muslim women who wear an Islamic head covering.

### Study design, sample, and recruitment

This research adopted Lieblich et al. (1998) narrative research approach, which provided the opportunity for women to share their experiences through storytelling. This methodological approach creates space for voices that are often silenced by dominant discourses (Wang & Geale, 2015). In doing so, narrative allows researchers to learn about the social and cultural world that shapes a person's identity and story (Lieblich et al., 1998). In the context of this research study, narrative methodology allowed the researchers to create a safe space for sharing one's experience through storytelling and shifts the power to the participant.

The study sample encompassed women who identified as Muslim, wore Islamic head coverings, and had healthcare encounters as a patient in Canada. The inclusion criteria also ensured participants understood and spoke English, were 18 years or older, were born in Canada or resided there for over 10 years. Institutional ethics approval was received for this study prior to recruitment, which included the use of virtual posters on social media platforms such as LinkedIn and Instagram.

### Data collection and data analysis

Data collection involved individual, semi-structured recorded interviews lasting 40 to 90 min beginning with a set of demographic questions. Samples of interview questions were: As a person who wears an Islamic head covering, can you tell me what these healthcare encounters as a patient were like for you? Did you have any challenging or positive experiences with your healthcare providers in the context of wearing an Islamic head covering? If you were talking directly to your healthcare providers, what would you like them to know about being a patient who wears an Islamic head covering? Despite the option of in-person interviews, all participants chose virtual interviews through Zoom. Researcher field notes captured non-verbal cues and additional insights during interviews.

**Figure 1.**
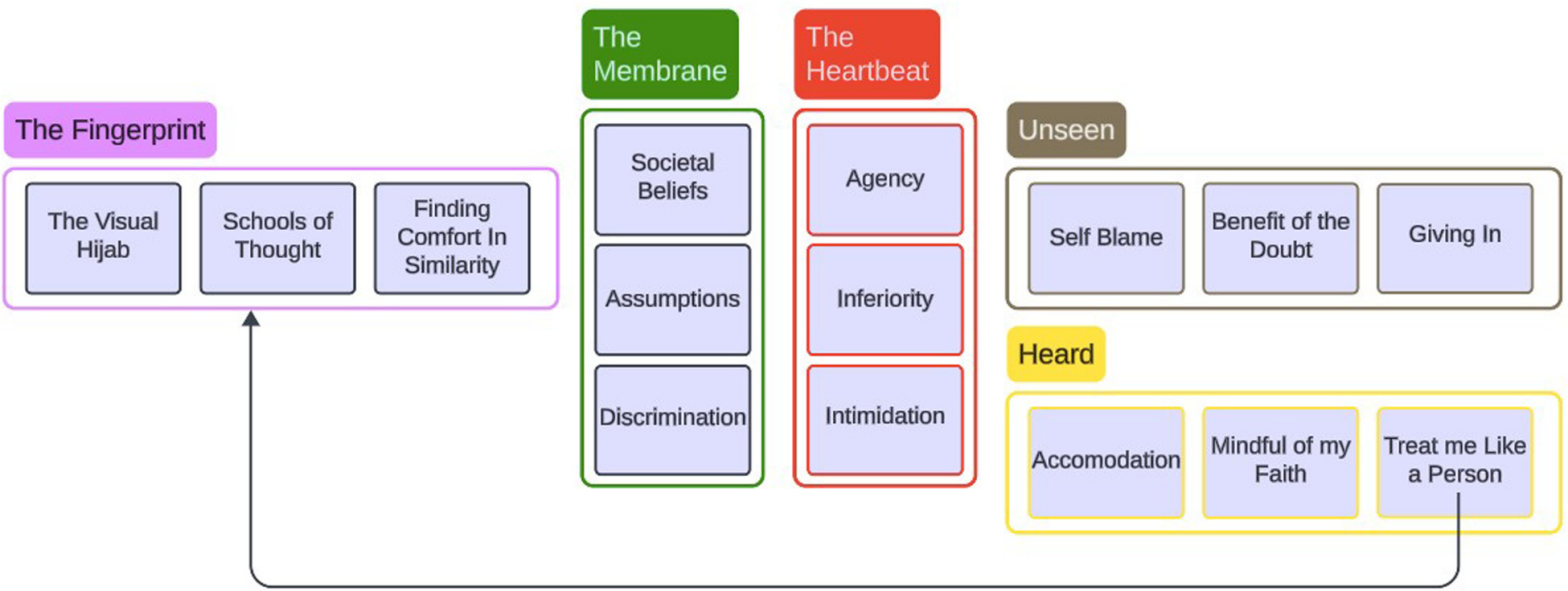
Overview of themes and codes.

The analysis framework for this study drew from Lieblich et al.'s (1998) narrative research approach, specifically categorical-content analysis and categorical-form analysis. Categorical-content analysis entailed an analysis of narrative content specifically linked to healthcare encounters among the participants. This process involved the meticulous development and refinement of codes, identifying and refining thematic elements inherent in the narratives. Subsequently, these refined codes were adeptly grouped into broader narrative themes, capturing the multifaceted aspects of the experiences shared (See Figure 1). In tandem with categorical-content analysis, categorical-form analysis delved deeper into the linguistic nuances shaping the narratives. This approach focused on exploring linguistic elements such as repetition, metaphors, and varying tenses employed within the narratives. By scrutinizing the intricate linguistic characteristics, this method extracted deeper insights, unveiling the subtleties and underlying emotions woven into the participants’ accounts. Participant excerpts will be used to highlight each code as is common in a narrative categorical analysis as opposed to a holistic story.

As the lead researcher, the first author conducted the recruitment and interviews and led the analytic process. Her dual identity as a Muslim woman who wears an Islamic head covering and a practicing nurse provided vital insight into this subject matter, and the healthcare encounters of similarly positioned women. Additionally, her positionality helped create a safe space to co-interrogate established knowledge and power relations with participants and deconstruct existing structures.

## Findings

The study included eight participants ranging in age from 20 to 53. Five were born in Canada, while the others migrated to Canada between 1975 and 2006. In terms of ethnic background, the participants represented diverse origins: four were South Asian, two were East African, one was North African, and one was Latin American. Ethnicities included Indian, Tanzanian, Argentinian, Moroccan, and Bengali. The age in which participants began wearing the Islamic head covering ranged from 10 years old to their late forties. The number of healthcare encounters ranged from 2 to 30 encounters in the past year.

Five central narrative themes—The Fingerprint, The Membrane, The Heartbeat, Unseen, and Heard—were constructed from the narratives, each interwoven with underlying codes that enrich and contextualize these thematic explorations (see Figure 1). Within these themes, a profound spectrum of experiences, emotions, and perceptions unfolds, painting a vivid portrait of the multifaceted realities encountered by Muslim women who wear Islamic head coverings in healthcare settings in Canada.

### Theme 1: The fingerprint

Within the theme “The Fingerprint”, these narratives delve into the intricate individualities inherent in these women's identities, unveiling the diversity of experiences in their healthcare encounters. This theme emerged from one participant's insightful comparison, likening Muslim women wearing Islamic head coverings to the uniqueness found in fingerprints: “everybody is different, just like your DNA. Just like your fingerprint” (Nora). This theme dissects the nuanced differences among Muslim women, highlighting the religious visibility of the Islamic head covering, the diversity in healthcare preferences based on differing schools of thought, and the tendency for some Muslim women to seek care from providers they perceive as ethnically or racially similar. The thematic exploration encompasses three codes: The Visual Hijab, Schools of Thought, and Finding Comfort in Similarity.

#### The Visual Hijab

Islamic head coverings significantly shape the identity of Muslim women, impacting how they are perceived and interact within healthcare contexts. Maryam, spoke on how she is not able to turn off her identity like her brother or husband are able to: “everyone will know I’m Muslim because of my headscarf … we become visual Muslims.” This perspective reveals an essential contrast: while males in her family can choose to conceal their religious identity, she cannot. This disparity establishes a unique identity, distinct from Muslim men and even other Muslim women who don’t wear an Islamic head covering. Unique to this group of women, Maryam's experience as a religiously visible Muslim woman illuminates how the Islamic head covering is a part of her identity that she, and other participants, cannot hide.

Wearing the Islamic head covering does not just affect external perceptions; it internally influences behaviors too. Another participant, Subhana, and her experience receiving a vaccination illustrates the internal shift generated by observing the hijab: “Before I wore the hijab, I was super comfortable, ‘Okay, you can see my upper arm.’ Now that I wear my hijab, I would ask for that accommodation for a woman to do it.” Her previous indifferences towards modesty or requesting a female provider shifted once she began wearing the hijab. This transformation suggests that her comfort levels with modesty and exposure altered in tandem with her adoption of the hijab.

#### Schools of thought

Varied understandings of Islamic principles, shaped by different *madhabs* (schools of Islamic jurisprudence), uniquely impact healthcare decisions. For example, when Subhana was asked what she would do if a female provider was not available, she expressed she would “postpone” her appointment, citing the “hadith” (e.g., religious text) regarding male-female interactions and how this was not permissible. Conversely, Maryam was very comfortable receiving healthcare services from a male provider, as it served a religious purpose and therefore, was Islamically permissible for her: “I’m allowed to show my hair. I’m allowed to show him my body part … it's not sinful on me.” The differences between these two women highlight the multiplicity in interpretations of religious texts among Muslim women, and how this may influence healthcare preferences differently.

#### Finding comfort in similarity

Finding Comfort in Similarity, a search for familiarity and understanding in healthcare encounters, illustrates the diverse preferences among Muslim women. For example, Maryam actively sought South Asian or racialized healthcare providers, seeking comfort in shared racial backgrounds: “[It was] more comfortable for me because I just didn’t feel different. … Maybe their parents are immigrants as well, maybe they can relate with my faith or my culture.” Similarly, Eva's “intentional” choice of seeking care from Muslim or South Asian healthcare providers is a way to obtain “comfort” because “it's someone who understands you” and she avoids potential biases due to cultural or religious unfamiliarity: “If I did go to someone who is definitely not well versed in my culture or religion, then I would feel that kind of bias … because of my hijab or my culture.” Subhana's preference for female practitioners reflects a broader collective sentiment among Muslim women to find comfort in similarity based on gender stating: “taking off like certain pieces of clothing in front of men, even just for medical reasons. It makes us uncomfortable … it’d be best for our comfort to have a female to look at us.” The differences in preferences among participants illuminates the variability that exists among this group of women. It emphasizes how external perceptions, internal changes, religious interpretations, and preferences intertwine to shape unique healthcare experiences.

### Theme 2: The membrane

Theme #2, titled “The Membrane”, illuminates how dominant societal discourses, entrenched in assumptions, and discriminatory attitudes infiltrate healthcare encounters for Muslim women who wear Islamic head coverings, often influencing the quality of care they receive. Just as a cell membrane selectively permits or denies matter, individuals filter prevailing discourses to construct their realities, profoundly influencing interactions with healthcare providers. “The Membrane” highlights the pressing need for a deeper comprehension of these dynamics, emphasizing the imperative to dismantle societal biases and presumptions within healthcare systems. The thematic exploration encompasses three codes: Societal Beliefs, Assumptions and Discrimination.

#### Societal beliefs

This code illuminates the substantial influence of societal perceptions and assumptions regarding Muslim women who wear Islamic head coverings on their healthcare encounters. Subhana remarked how society tends to believe that Muslim women seem to be oppressed and helpless, because their God doesn’t allow for [them to do] anything”. This highlights a prevalent stereotype that portrays Muslim women as inherently oppressed due to their faith practices. Such assumptions are entangled within power dynamics perpetuated by media representations. Eva indicated how it's “the media” that reinforces these “negative thoughts about Islam” and people “become ignorant because you don’t educate … or be unbiased … [you] use those people's opinions to make your own.” Although education can tackle healthcare providers’ societal beliefs, Amina's insights into educational materials underscores the reality of existing material related to culture and/or religion as she says: “It's off to the side. You ‘re [going to] ignore that little orange box because you need to get to the meat of the information”. Her observation elucidates how educational materials often sideline crucial cultural considerations, leading to misinformed assumptions derived from media representations influencing healthcare interactions. In essence, these narratives reveal the pervasive impact of societal beliefs, often distorted by media representations, shaping multifaceted and biased healthcare encounters for this group of women.

#### Assumptions

This code delves into the prejudiced beliefs imposed upon Muslim women who wear Islamic head coverings. These assumptions, rooted in societal biases and often fueled by media portrayals, act as barriers, affecting the quality of healthcare encounters. Ayesha's experience with a physician who requested she take off her hijab for an ear assessment exemplifies the impact of societal assumptions. When she refused, it “felt like he thought I was being extreme”. Ayesha's account underscores the misinterpretation of her religious observance as an extreme stance, highlighting the conflict between her religious beliefs and the healthcare provider's assumptions. The connotations associated with the linguistic usage of “extreme” is often associated with Muslims who are radicalized. Similarly Amina spoke about “the assumption” that Muslim women are “not going to talk that much … it's just going to be simple, quick, and easy and out”. Amina's insight exposes how assumptions hinder healthcare providers from engaging with Muslim women effectively, leading to rushed interactions and disregarding their individual needs. Oftentimes, there are assumptions surrounding sexual health. Fathima comments that they assume that “just you’re Muslim, you don’t have sex. But why are you making that assumption?” She elaborated that “it really is damaging because then kids have nowhere to go … pushes them away from getting the help they receive.” These narratives emphasize how assumptions ultimately perpetuate biased care and hinder patient-provider communication.

#### Discrimination

This code elucidates how Muslim women who wear Islamic head coverings face discrimination within healthcare encounters because of societal biases and assumptions. For example, while Maryam herself was discriminated against, she also noticed contrasting treatment between herself and her sister-in-law at the Emergency Department. She shared, “my sister-in-law, wearing the niqab, was told “You can’t have anyone here”. There was a part of me where I was like, maybe it's because [she's] wearing what [she's] wearing”. This implies differential treatment based on appearance, as Maryam was told she would be allowed to have visitors. Ultimately, this reflects potential biases against Muslim women who wear an Islamic head covering. Ayesha, who converted to Islam later on in life, also shared she experienced discrimination more after she became Muslim. She noted a change in how her privacy was respected after adopting Islam: “Doctors respected my privacy more before I was Muslim … before I was Muslim, I wasn’t dressing this modestly, so it was strange to me”. Her observation highlights the implicit discrimination faced by Muslim women based on religious appearance, impacting their healthcare encounters. Another participant indicated how a white physician “didn’t believe anything I had to say … there's something wrong with me, and he wouldn’t believe me.” These excerpts highlight the serious impact of discrimination on these women's healthcare needs.

### Theme 3: The heartbeat

Within Theme #3, “The Heartbeat”, the experiences of Muslim women who wear Islamic head coverings unravel as a complex interplay of emotions. This thematic exploration delves into primeval responses to codes in Theme #2, akin to a metaphorical heartbeat, resonating with the diverse facets encountered during care. It encapsulates not only the physical reactions but also emotional reactions—sometimes pulsating with resolve when practicing agency, and other times, racing with distress when experiencing intimidation and inferiority. The thematic exploration encompasses three codes: Agency, Inferiority, and Intimidation.

#### Agency

The code Agency refers to how Muslim women exerted control over dominant discourses about them as patients. Ayesha exemplifies this by asserting herself when a male physician instructed her to lift her shirt for examination. She bravely responded with, “Sorry, I just don’t feel comfortable with you touching me. Is there a female doctor here that could see me possibly?” Her agency defied societal norms and religious beliefs, illustrating resistance against a male figure's authority. Conversely, Fathima demonstrated agency through silence when male physicians entered her room during an MRI. She explained, “I know that if I had spoken up, the attention would have been on me. If I ignored it, he would have walked away, and because I wasn’t covered, I wanted the minimal amount of attention.” Her deliberate choice to remain quiet was an act of control, strategically avoiding unwanted attention and maintaining a sense of power over the situation. These examples illuminate how Muslim women navigate power dynamics in healthcare by either challenging authority or strategically choosing silence to assert their agency in varying situations.

#### Inferiority

In the context of healthcare encounters, the code Inferiority encapsulates the feelings of inadequacy experienced by Muslim women wearing Islamic head coverings. Eva explicitly stated “feeling inferior” when being cared for by providers who were non-Muslim. Fathima shared her experiences where she felt her doctor's discomfort and reluctance to engage fully due to her hijab and Muslim identity. She noted, “certain white male doctors wouldn’t believe [her] … a part of it has to be [her] being a Muslim, because you can tell that [the physician is] uncomfortable with [her]. With [her] hijab, [her] presence, and [the physician] wouldn’t even look at [her] properly. Wouldn’t even say [her] name.” By specifying the race and gender of her white and male doctors, Fathima underlines societal norms and power structures linked to race and gender, perpetuating the notion of superiority associated with certain demographics. Fathima's encounter also illuminates the complex interplay between race, religion, age, and patient vulnerability, compounding the sense of inadequacy experienced by these women during their healthcare encounters.

#### Intimidation

The code Intimidation captures the fear experienced by Muslim women wearing Islamic head coverings due to behaviors exhibited by healthcare team members. Maryam found it “intimidating” when healthcare providers questioned her symptoms: “It was done in a very disrespectful way ‘Explain your pain’ … I’m not lying.” She elaborated how she has “seen how people talk to other people … If that conversation was with a person who is not of colour, it could have been like that for [her].” She sensed the disrespect and also feared not being believed, amplifying her distress due to severe pain and the perception of disparate treatment based on her appearance and race. This underlines societal norms and prejudices influencing the care received by Muslim women, reflecting a sense of subordination and fear of inadequate treatment.

Similarly, Fathima's past experience led to significant anxiety about visiting doctors: “I developed a lot of anxiety going to the doctor and now I don’t go to the doctor. It takes a lot for my friends to help me to go to the doctors, because I have a lot of anxiety.” Her apprehension stemmed from a strong belief that her concerns would not be taken seriously, indicating the substantial psychological impact of intimidation. Fathima's anxiety, quantified as significant through her repeated emphasis on “a lot” underscores the profound psychological toll imposed upon her within healthcare encounters. This also demonstrates the power imbalance where providers hold the authority to discredit the experiences of Muslim women in head coverings, thereby limiting their access to adequate and equitable care.

### Theme 4: The unseen

In Theme #4, “Unseen”, the focus is on the absence of a perceptual dimension (whereas theme #1-3 focused on physical body parts). This theme unveils a distressing reality where the visibility of Muslim women who wear an Islamic head covering paradoxically render themselves as unseen in the context of their healthcare encounters. This theme sheds light on the struggle to reconcile the visibility of their religious expression with the lack of acknowledgment and support, underpinning the emotional turmoil and their unmet needs. The codes subsumed under this theme are Self-Blame, Benefit of the Doubt, and Giving In.

#### Self-Blame

This code encompasses Muslim women wearing Islamic head coverings taking onus of negative healthcare outcomes. Despite requesting a female gynecologist and having her doctor reinforce the request, Maryam still blamed herself as she stated, “Did I ever [follow up and] ask? I didn’t. I blame myself too.” Ayesha was asked if she ever spoke up about her negative encounters, she stated suppressing upsetting encounters even though she acknowledges the potential for change if she did speak up: “Even though maybe I should … try and make a change for other hijabi women.” Both women reflect with self-blame, with Maryam questioning herself and Ayesha feeling responsible for systemic issues. These experiences illustrate the struggle of self-blame within healthcare structures and the realization of invisibility experienced by Muslim women.

#### Benefit of the doubt

This code reveals Muslim women who wear an Islamic head covering giving healthcare providers the benefit of the doubt. Maryam attributes a white healthcare provider's attitude to the timing of the night: “[she] looked really annoyed … [I] can’t really blame anyone. It was like three, four AM” despite the normalcy in attitude this provider exemplified towards other patients. She refrains from attributing annoyance to discrimination, and instead, aligns it with existing power structures. Similarly, Fathima speculates that a female MRI technician might have made “a suggestion”, but didn’t say it was “mandatory” for male providers to not walk in the room. She states, “I don’t know what actually happened. But I know my privacy was breached,” but refrains from accusing the provider of an intentional privacy breach. Both women avoid assigning negative intentions to provider's actions, yet their encounters hint at such implications.

#### Giving in

Under this code, Muslim women who wear Islamic head coverings are seen acquiescing to healthcare situations or provider advice, sometimes at the expense of their religious needs remaining unmet. Maryam described a sense of resignation when she states, “Sometimes we just give in … it's just going to be easier.” Her use of ‘we’ implies shared experiences with other Muslim women who wear Islamic head coverings. Similarly, Ayesha requested “a private room” during “blood work” but “they just refused, and there was nothing really I could do because I was not feeling well already.” Despite her discomfort and the feeling of vulnerability, she complied. The power imbalance between herself and the healthcare system became apparent. Fathima exemplified the taxing nature of advocating for oneself: “I stopped with the doctors because I was so exhausted.” Her experience highlights the challenges—exhaustion and anxiety—that hinder constant assertion of needs. These encounters reflect the struggle against oppressive norms and power structures within healthcare settings, where the women oscillate between practicing agency and yielding to existing power imbalances.

### Theme 5: Heard

Theme #5, “Hear”, illuminates the narratives of Muslim women who wear Islamic head coverings and their encounters where they feel validated and recognized by healthcare providers. This theme diverges from Theme #4 by spotlighting instances where women feel listened to and acknowledged. There are three codes within this theme: Mindful of my Faith, Accommodation, and Treat me Like a Person.

#### Mindful of my faith

This code embodies the experiences of Muslim women who wear Islamic head coverings feeling validated by healthcare providers who are attentive to their religious needs. In this context, providers who proactively acknowledge and respect the religious beliefs of these women contribute to their sense of being heard and understood. Maryam shared an account about a skin issue on her head describing her “Jewish” physician as “very mindful of my faith.” She recalled him stating “You know what, “I’m gonna refer you to a female dermatologist … it's not necessary for me to look at your hair.” Despite following different religions, Maryam's physician exhibited a deep mindfulness of her Islamic faith, which influenced his medical approach. His decision to refer her to a female dermatologist, despite Maryam's willingness to show her hair for examination, illustrated his respect for her religious practices and family traditions and made her feel acknowledged and respected, contributing to her sense of being “Heard”. This code exemplifies the significance of practitioners actively acknowledging and respecting the diverse religious identities of their patients, ultimately fostering a sense of being heard and understood within healthcare encounters.

#### Accommodation

The code “Accomodation” captures instances where Muslim women who wear Islamic head coverings make specific requests aligned with their religious beliefs, seeking acknowledgment and acceptance of their needs within healthcare encounters. Subhana's experience receiving her COVID-19 vaccine exemplifies the responsiveness of some healthcare providers to accommodate religious preferences. She shared she “requested to go to the back room, and go with the female nurse, and they … didn’t hesitate. They were very accommodating.” Her request for both a private space and specific provider was honored. Subhana's positive encounter portrays a scenario where her voice was acknowledged, indicating a significant aspect of feeling heard and respected within the healthcare environment.

#### Treat me like a person

*“*Treat me Like a Person” illuminates Muslim women's’ longing to shed the weight of stereotypes and biases that exist towards them by advocating for a simple, yet profound, need: to be regarded as individuals and treated like a person. Maryam's moving request, “Just treat me like a person … how you would treat any other person” echoes the shared sentiment among these women, stemming from past experiences where their humanity was eclipsed. Eva and Fathima both emphasized the need to transcend misconceptions and biases inherent in medical contexts. Eva asserts the depth of her individuality, advocating for recognition beyond visible differences when she shared, “I wish [healthcare providers] understood or realized that we’re just like everyone else. We may have a different ethnicity. And we show our religion. But that doesn’t change what's under the head covering. We’re still a person”. Fathima similarly shared, “Whenever I’m in the healthcare system, they look at me as a body versus a person”. It is apparent that Fathima's existence feels depersonalized, and she seeks to be treated like a person as opposed to a mere body.

Their collective narrative resonates with Nora's analogy to fingerprints, emphasizing the unique nature of each person's story. The call for healthcare providers to actively engage with these narratives and understand their diverse experiences is a call for genuine and empathetic care. In essence, Theme #5 transcends the desire for recognition—it stands as a testament to the imperative need to not just witness but actively listen and acknowledge the distinctive experiences and needs of Muslim women who wear Islamic head coverings. It advocates for an inclusive healthcare environment where Muslim women's stories are *heard*.

## Discussion

In this study, we aimed to explore how Muslim women who wear Islamic head coverings narrate their experiences of healthcare encounters as a patient in Canada and how discourse shapes their narratives. In answering these questions, we found that their stories illuminate a transformative narrative that unveils layers of complexity and challenges prevalent perceptions. The lead researcher in this storied and transformative journey found herself akin to a sculptor who crafts a narrative by sculpting using a block of clay. Much like a plain block of clay, care towards these women was often standardized and uniform. Through their rich narratives, it became apparent that this group of women sought care that is individualized.

Initially, these women's encounters were perceived to be generalized, as their unique healthcare preferences and needs were obscured. In such instances, healthcare professionals adopted a generalized one-size-fits-all approach when caring for Muslim women who wear an Islamic head covering. As a result, there is apparent oversight regarding the diverse preferences among with group of women.

The generalization of care towards this group of women aligns with Razack's (2008) “racial thinking”. When a group of individuals belong to a community that is not of the dominant culture, as is the case for Muslim women who wear an Islamic head covering, the individuality and differences that exist among the group are often overlooked; this is consistent with “racial thinking” outlined by Razack. As a result, the care provided to this group of women is homogenized, as exemplified through the participants’ stories. Overall, this group of women are often seen as a homogenous group defined by their head coverings, simplifying their complex and intricate identities to a single visible marker.

The homogenization of this group of women is further reinforced by prevailing social discourses and stereotypes about this group of women. Dominant social discourses, such as the media, perpetuate certain stereotypes and misconceptions about Muslim women who wear Islamic head coverings (Hoodfar, 2003), which further cements the portrayal of Muslim women who wear an Islamic head covering as a homogenous group. The visibility of this group of women makes them subject to Rahmath et al.'s (2016) notion of being the “other”, making them more susceptible to such societal stereotypes in comparison to Muslim women who may not wear an Islamic head covering or Muslim men. As exemplified by various narratives shared by participants, healthcare providers may take a homogenized approach by relying on dominant discourses when providing care, as opposed to engaging with and responding to the individual and diverse needs that exist among this group of women. Additionally, Siraj (2011) outlines that religious preferences are related to individual interpretations of Islamic law. This is consistent with the varying healthcare preferences among participants. Ultimately, these narratives emphasize the urgency to move away from homogenization and deconstruct the dominant discourses about Muslim women who wear an Islamic head covering.

A poststructural lens, deconstructs the homogeneity of healthcare encounters for this group of women, by illuminating different tenets (such as race, age, etc.) of one's identity that impacts the care this group of women receive. The dissection of narratives illuminated the diversity of identities, preferences and experiences which counters the initial generalization among this group of women. Deconstructing women's narratives made apparent the power imbalances that exist between female Muslim patients and healthcare providers, as noted in other studies (Simpson & Carter, 2008). Specifically, healthcare providers, who hold significant power in society because of their education and their occupation, were described to be dismissive or disengaged towards many participants.

Building on this analysis, Murrar et al. (2024) further elaborate on the complexities of discrimination within healthcare encounters, highlighting how ethnic and racial identities intersect with religious ones to shape the experiences of this group of women. Specifically, Murrar et al. outlines how race also contributes to the way in which this group of women are treated, as they report that Arabs and South Asians reported less discrimination than African American women in their study. Echoing such findings, a Hispanic participant in our study expressed how she experienced discrimination during her in-person appointments, but this was not the case for appointments that took place over the phone. She appoints this to her non-Muslim sounding name, which masks her religious identity in non-visual interactions.

Age also emerged as a factor influencing healthcare encounters, with older participants sharing a generally more positive tone about their healthcare encounters. This may be attributed to second-generation immigrants facing more obstacles from perceived discrimination due to their deeper engagement in identity-forming processes as noted by Giuliani et al. (2018). In contrast, first generation-immigrants maintain stronger connections to their cultural roots which buffer their exposure to negative experiences (Inman, 2006). These varied encounters and realities underscore the necessity of tailored care, accommodating the diverse perspectives and experiences of Muslim women who wear an Islamic head covering within healthcare settings.

It is apparent that factors such as power, race, and generational status influence both the quality of care received by Muslim women, as well as how healthcare encounters are perceived. This highlights the need for a healthcare system that looks beyond the physical marker that is the Islamic head covering, and instead a healthcare system that adopts an approach that acknowledges and actively incorporates those various factors that impact the healthcare preferences and needs of Muslim women who wear an Islamic head covering. The diverse experiences across participants underscores the ultimate need for healthcare systems to move beyond a superficial understanding about this group women and to instead, engage deeply in stories and a contextual understanding of healthcare preferences and needs.

At the pinnacle of this transformative journey stands the artistry of tailored care, which represents a shift from homogenized to individualized care and recognizing that every Muslim woman who wears an Islamic head covering is shaped by their own unique experiences. Overt acts of discrimination towards Muslim women are prevalent in healthcare, whether in the form of a healthcare provider's reluctance to provide care, making offensive comments, or rushing care (Nur, 2014; Zine, 2006). This was reinforced by the narratives shared by participants, through both verbal and non-verbal communication. Practicing the artistry of tailored care involves encouraging healthcare providers to reflect on how dominant ideologies shape their actions when providing care to this group of women, reflecting on biases they may hold, addressing such biases and then intervening accordingly.

While there may be structural limitations that are difficult to address or accommodate, Alzghoul et al. (2021) outlines the importance of practical and effective solutions to uphold Muslim womens’ modesty. While a one-size-fits-all garment (i.e., the gown worn for an MRI) is desirable from an economic perspective, healthcare providers can consider providing alternatives such as pyjama pants to cover their legs or allowing clients to wear their own clothing where appropriate.

Our study also illuminated the deficiencies in current healthcare education, with participants detailing existing sidelined educational materials on cultural training is not sufficient. Specifically, our research echoed Stone and Moskowitz (2011) work detailing how current training for healthcare providers is superficial and performative, with cultural considerations in the margins of textbooks and ineffective implications of contemporary training programs. Information in training materials is often homogenized, and encouraged to be applied to all Muslim women who wear an Islamic head covering. However, the artistry of tailored care requires understanding the unique and varied preferences among this group of women and disrupting the notion of homogeneity. Poststructuralism proposes the multiplicity of truths and there is a dire need for Muslim women to share their stories and bring to surface their own truths that will impact the care they receive in a positive and meaningful way. The artistry of tailored care involves a genuine understanding of this group of women to ultimately defy the homogenization of care.

Genuinely understanding this group of women signifies a fundamental reimagining of healthcare encounters. It calls for the dismantling of homogenized perceptions in nursing practice, the honoring of individual identities and preferences, and the fostering of inclusive, respectful healthcare environments. Nurse and healthcare leaders should actively consult Muslim women who wear Islamic head coverings to ensure that policies are inclusive and reflect the diverse preferences among this group of women. In daily practice, it is vital to provide practical accommodations to foster a more inclusive environment, such as providing appropriate clothing options.

Upholding and supporting individual healthcare decision-making, especially in consideration of topics pertaining to sexual health, can help establish trust and respect between the healthcare provider and the client. It is vital to ensure self-reflection remains a keystone in nursing education in which students actively scrutinize their beliefs and biases in order to nurture a more inclusive and culturally adept workforce. Moreover, training programs in both academic and healthcare institutions should embed firsthand accounts of patients and extensively cover topics such as microaggressions, diverse healthcare preferences, and methodologies. These approaches empower students and nurses to navigate the intricacies of providing respectful and culturally safe care to this specific demographic.

## Recommendations

Healthcare providers should be mindful of practices that respect cultural practices of Muslim women who wear an Islamic head covering. This includes efforts to ensure access to female providers when requested, accommodating needs for privacy and modesty (including offering modest attire when needed or draping the patient appropriately) and promoting an environment where the patient feels comfortable voicing their healthcare preferences and needs. Policies should promote inclusivity and prevent discrimination within healthcare settings, ensuring that religious practices are respected. Muslim women who wear Islamic head coverings should be consulted where appropriate, and considerations must be made towards the individual differences that may exists among this group of women. It is important that nursing students are supported to critically examine their biases and understand how personal beliefs might impact the care provided to this group of women. Emphasizing a cultural humility approach in nursing education will better positions students to be open to learning about patient needs. Additionally, including the stories of Muslim women within nursing curriculum will allow students to learn *from* patients, as opposed to learn *about* patients.

## Strengths and limitations

A rigorous and reflexive approach to this research enhanced the richness of these findings. As a team of Muslim and non-Muslim researchers, we engaged in iterative, critical, and dialogical discussions, which deepened and strengthened the analytical process. Use of a poststructuralist theoretical lens also deepened the analysis beyond mere description in which we were able to critically examine how discourse shaped narratives. Although not incorporated into this manuscript, the first author constructed “Thank You” poems following each interview, as a reflective exercise that allowed a deeper appreciation of participants’ stories. Despite a small number of concepts drawn from eight interviews, this study obtained rich narrative data to ultimately achieve a deep and thorough understanding of how existing ideologies shape the healthcare experiences of Muslim women who wear an Islamic head covering. This study benefitted from the diversity that existed among participants, both regarding racial/ethnic backgrounds as well as age. This allowed the researchers to capture both the diverging and converging experiential narrative accounts of Muslim women who wear an Islamic head covering and their healthcare encounters in Canada.

While diverse thematic analysis and engaging dialogues were embraced, further diversification of racial backgrounds among participants could enrich its breadth (in particular, the inclusion of Black Muslim women). A full and meaningful picture was created with converging and diverging experiential narrative accounts, yet it highlighted potential areas for deeper exploration. In consideration of future research, the exploration of how age shapes healthcare experiences among Muslim women who wear an Islamic head covering can drive tailored interventions across all age groups. Although this research focused mainly on primary and emergency care, investigating other settings (i.e., rehabilitation centers and critical care units) could reveal specific challenges to such locations. Additionally, the integration of other theories such as intersectionality or a critical feminist perspective offers a different, but important understanding of the encounters this group of women had. Ultimately, these approaches would deepen insights further and optimize the care that is provided to this group of women.

## Conclusion

The core issue is evident: the healthcare encounters of Muslim women who wear Islamic head coverings must transcend uniformity. These women deserve care that is personalized, inclusive, and respectful, acknowledging their individual identities, preferences, and diverse experiences. Embracing the Artistry of Tailored Care is pivotal to cultivate empathetic, considerate, and fair healthcare for these women within the Canadian healthcare system. The Artistry of Tailored Care signifies a shift from standardized to personalized, patient-centered healthcare. It champions the uniqueness of each woman and implores healthcare providers to confront implicit biases, address discriminative behaviors, and navigate complex limitations. This artistry necessitates a reimagination of healthcare education, transcending superficial cultural competence sessions to foster authentic understandings that honor the diversity among these women. It is a plea for systemic transformation, recognizing and responding to the sophisticated narratives and needs within this community, ultimately ensuring that every Muslim woman who wears an Islamic head covering is *treated like a person.*
